# Molecular characterization and copy number of *SMN1*, *SMN2* and *NAIP* in Chinese patients with spinal muscular atrophy and unrelated healthy controls

**DOI:** 10.1186/s12891-015-0457-x

**Published:** 2015-02-07

**Authors:** Ping Fang, Liang Li, Jian Zeng, Wan-Jun Zhou, Wei-Qing Wu, Ze-Yan Zhong, Ti-Zhen Yan, Jian-Sheng Xie, Jing Huang, Li Lin, Ying Zhao, Xiang-Min Xu

**Affiliations:** Department of Medical Genetics, School of Basic Medical Sciences, Southern Medical University, Avenue North1838, Guangzhou, Guangdong People’s Republic of China; Department of Clinical Laboratory, The Fuzhou General Hospital, Nanjing Military Command, Fuzhou, Fujian People’s Republic of China; Prenatal Diagnosis Center, Shenzhen Maternity and Child Healthcare Hospital, Shenzhen, Guangdong People’s Republic of China; Liuzhou Key Laboratory of birth defects prevention and control, Liuzhou Municipal Maternity and Child Healthcare Hospital, Liuzhou, Guangxi People’s Republic of China; The Third Affiliated Hospital of Southern Medical University, Guangzhou, Guangdong People’s Republic of China; Prenatal Diagnostic Center, Dongguan Maternal and Children Health Hospital, Dongguan, Guangdong People’s Republic of China

**Keywords:** Chinese, MLPA, SMA, Gene copy number

## Abstract

**Background:**

Spinal muscular atrophy (SMA) is caused by *SMN1* dysfunction, and the copy number of *SMN2* and *NAIP* can modify the phenotype of SMA. The aim of this study was to analyze the copy numbers and gene structures of SMA-related genes in Chinese SMA patients and unrelated healthy controls.

**Methods:**

Forty-two Chinese SMA patients and two hundred and twelve unrelated healthy Chinese individuals were enrolled in our study. The copy numbers and gene structures of SMA-related genes were measured by MLPA assay.

**Results:**

We identified a homozygous deletion of *SMN1* in exons 7 and 8 in 37 of 42 patients (88.1%); the other 5 SMA patients (11.9%) had a single copy of *SMN1* exon 8. The proportions of the 212 unrelated healthy controls with different copy numbers for the normal *SMN1* gene were 1 copy in 4 individuals (1.9%), 2 copies in 203 (95.7%) and 3 copies in 5 (2.4%). Three hybrid *SMN* genes and five genes that lack partial sequences were found in SMA patients and healthy controls. Distributions of copy numbers for normal *SMN2* and *NAIP* were significantly different (*P* < 0.001) in people with and without SMA.

**Conclusion:**

The copy numbers and gene structures of SMA-related genes were different in Chinese SMA patients and healthy controls.

## Background

Spinal muscular atrophy (SMA) is one of the most common autosomal recessive diseases and is characterized by degeneration of spinal cord motor neurons, atrophy of skeletal muscles, and generalized weakness [1]. The incidence of SMA is approximately 1/6,000 to 1/10,000 live births, and the carrier frequency is about 1/42 in the Chinese population [2]. SMA is divided into four clinical types according to age of onset and achieved motor function: (1) severe type I; (2) intermediate type II; (3) mild type III; and (4) adult-onset type IV.

SMA is caused by the dysfunction of the survival motor neuron (*SMN*) gene on chromosome 5q13.2. The two versions of *SMN*, *SMN1* and *SMN2*, differ by only five nucleotides. *SMN1* produces a full-length transcript that encodes functional SMN protein. About 94% of SMA patients have a homozygous deletion of *SMN1* exon 7. *SMN1* can be absent because of deletion or SMN1-to-SMN2 conversion [3]. A single nucleotide transition in *SMN2* exon 7 (c.840C > T) relative to *SMN1* causes most of the *SMN2* pre-mRNA to lack exon 7 and encode nonfunctional SMNΔ7 protein [4]. However, about 10% of *SMN2* pre-mRNA is normal and can be translated into full-length SMN protein. *SMN2* partially functionally compensates an *SMN1* homozygous deletion [5]. Thus, the *SMN2* copy number influences SMA severity. In addition to the *SMN* genes, the neuronal apoptosis inhibitory protein (*NAIP*) gene, also located at chromosome 5q13.2, is an SMA disease-related gene. The copy number of *NAIP* is reported to be correlated with SMA severity. SMA patients with fewer copies of *NAIP* have more severe phenotypes than patients with more copies of *NAIP* [6,7]. Moreover, both *SMN2* and *NAIP* copy numbers were associated with the onset age, risk of death and survival probability of SMA patients [8]. Therefore, elucidating the gene copy numbers and structures of *SMN1*, *SMN2* and *NAIP* is important for analyzing the molecular mechanism of SMA and for SMA clinical diagnosis.

The *SMN1*, *SMN2* and *NAIP* genes are all located at 5q13.2, an unstable chromosomal region that is prone to deletion, duplication, and gene conversion. Copy numbers of *SMN1* and *SMN2* vary in humans. In Chinese populations, up to 4 *SMN1* or *SMN2* genes are reported in both healthy people and SMA patients [2,9]. In addition, *SMN* gene deletions and rearrangements are found in different populations [6,7,10]. Detecting copies and gene structures of SMA-related genes is difficult because of the high homology between *SMN1* and *SMN2*. However, the multiple ligation-dependent probe amplification (MLPA) assay has been established for detecting deletions and duplications of SMA-related genes [11,12].

To determine the copy numbers and structures of SMA-related genes in a Chinese population, we analyzed *SMN1*, *SMN2* and *NAIP* in 42 Chinese SMA patients and 212 Chinese healthy individuals using MLPA assays.

## Methods

### Population and patient samples

A total of 212 peripheral blood samples were obtained from unrelated healthy adults (108 men and 104 women) at the Third Affiliated Hospital of Southern Medical University. In addition, we enrolled 42 unrelated SMA patients from four hospitals (18 patients from the Fuzhou General Hospital of Nanjing Military Command, 18 from the Shenzhen Maternity and Child Healthcare Hospital, 4 from the Liuzhou Municipal Maternity and Child Healthcare Hospital, and 2 from the Prenatal Diagnosis Center of Dongguan Maternal and Child Health Hospital). All SMA patients were confirmed by MLPA assay to have a homozygous deletion of *SMN1* gene exon 7. All healthy individuals and SMA patients were of Han ethnicity.

### Ethical approval

Ethical approval for this study has been obtained from the Ethical Committee of the Southern Medical University as well as from partners including the Ethics Committee of the Third Affiliated Hospital of the Southern Medical University, the Ethics Committee of Fuzhou General Hospital of Nanjing Military Command, the Ethics Committee of Shenzhen Maternal and Child Health Care Hospital, the Ethics Committee of Liuzhou Maternal and Child Health Care Hospital and the Ethics Committee of Reproductive Medical Center of Dongguan Maternal and Child Health Care Hospital. Written informed consent was obtained from all participants or guardians prior to the study.

### MLPA technique

**Figure 1:**
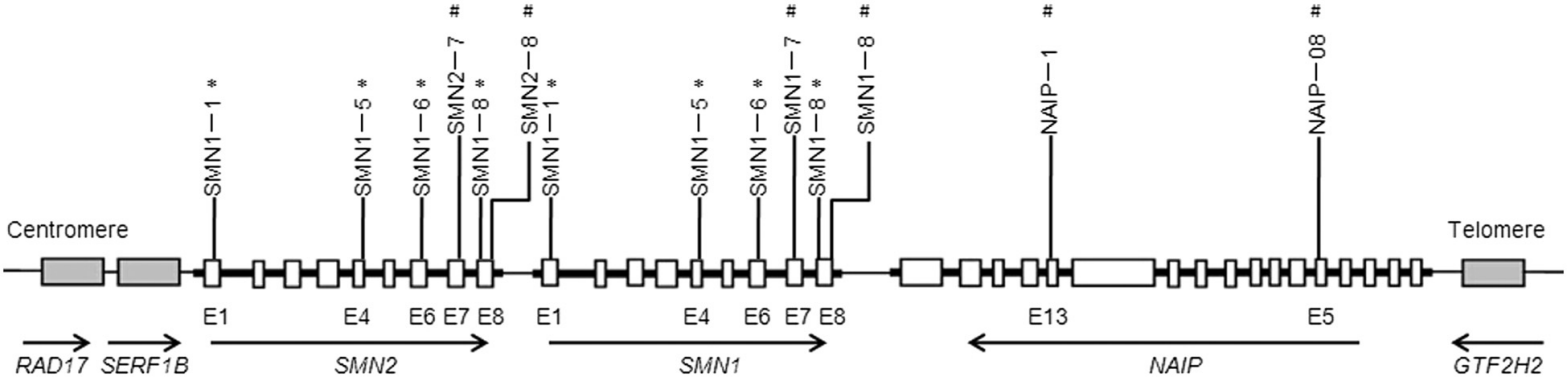
**Schematic representation of SMA-related genes in MLPA assays.** Gray, *RAD17*, *SERF1B* and *GTF2H2*, near the disease-determining gene. Arrows, relative position and 5′-to-3′ direction of SMA-related genes. Blank boxes, exons (E). Important MLPA probes are above related genes. *, probes complementary to common regions in exons 1, 4, 6, 8 of either *SMN1* or *SMN2*; #, probes specific for *SMN* or *NAIP* genes.

Human DNA was extracted from leukocytes in peripheral blood using a standard phenol/chloroform method. *SMN1*, *SMN2* and *NAIP* copy numbers were determined by an MLPA technique using SALSA P021-A2 SMA kits (MRC-Holland, Amsterdam, Netherlands) that amplify 37 regions throughout the genome for products between 140 and 463 bp. Of the amplified regions, 15 were target sequences at the SMA locus on 5q13.2 and 22 were reference sequences. Ten probes were complementary to *SMN1*, *SMN2* and *NAIP* sequences. Because of the sequence similarity of *SMN1* and *SMN2*, probes SMN1-1^*^, SMN1-5^*^, SMN1-6^*^ and SMN1-8^*^ were complementary to common regions in exons 1, 4, 6, 8 of either *SMN1* or *SMN2*. Probes SMN1-7^#^ and SMN1-8^#^ were specific for *SMN1* in exons 7 and 8. Probes SMN2-7^#^ and SMN2-8^#^ were specific for *SMN2* in exons 7 and 8. Probes NAIP-1^#^ and NAIP-08^#^ were specific for *NAIP* in exons 13 and 5 (Figure 1). MLPA assays are a multiplex PCR strategy with four steps: DNA denaturation and probe hybridization, ligation of two probes, PCR of ligated probes, and separation of amplified fragments and data analysis. All experimental procedures were carried out according to kit instructions. MLPA products were separated and quantified by capillary electrophoresis using an ABI 3130XL Genetic Analyzer with LIZ 500 as the internal size standard. Data were analyzed with the GeneMapper software v3.2 package (Applied Biosystems, Foster city, CA). Intrasample normalization was by dividing the peak area of each probe’s amplification product by the total area of reference probes only. Intersample normalization was by dividing the intranormalized probe ratio for a sample by the average intranormalized probe ratio of two reference samples. Reference samples had two copies of *SMN1*, *SMN2* and *NAIP* genes. Intervals for estimating the copy number of each probe were defined according to the predefined standards of Alías et al. [13].

### Statistical analysis

The distributions of samples with different *SMN2* and *NAIP* copy numbers from SMA patients and healthy individuals were compared using the Mann–Whitney *U* test. A p-value of less than 0.05 was considered statistically significant. All statistical analyses used the SPSS software package (version 13.0, SPSS Inc., Chicago, IL).

## Results

### Gene copy number and structure in SMA patients

**Figure 2:**
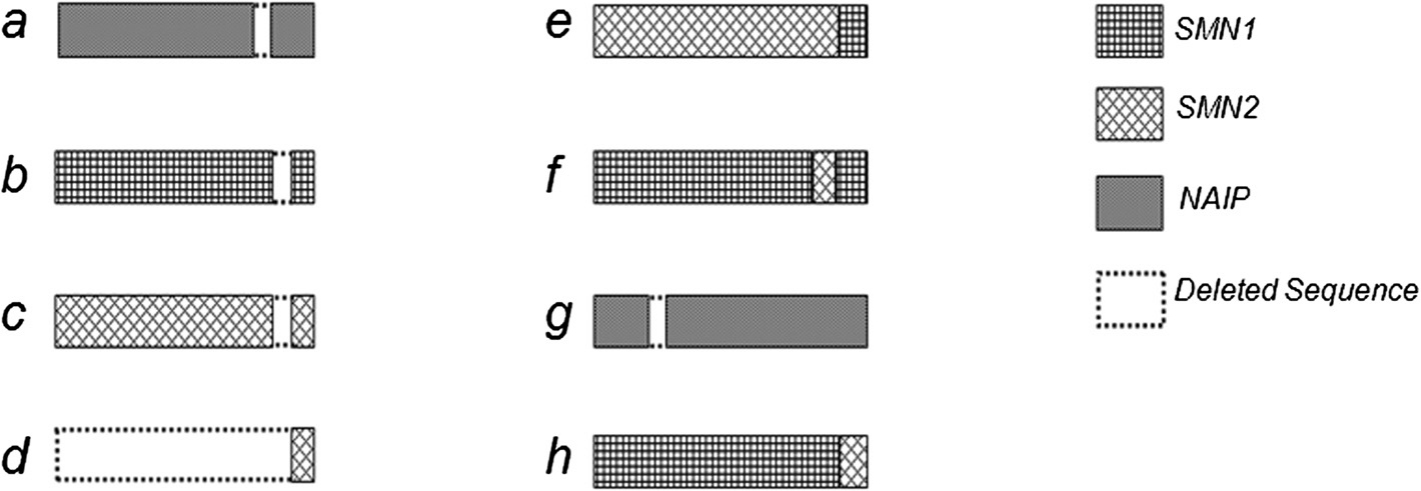
**Gene structures inferred from MLPA results. (** ***a*** **)** *NAIP* gene lacking exon 5; **(** ***b*** **)** *SMN1* gene lacking exon 7; **(** ***c*** **)** *SMN2* gene lacking exon 7; **(** ***d*** **)** *SMN2* gene lacking exon 1 to exon 7; **(** ***e*** **)** Hybrid *SMN* gene in which exon 8 of *SMN2* was converted to exon 8 of *SMN1*; **(** ***f*** **)** Hybrid *SMN* gene in which exon 7 of *SMN1* was converted to exon 7 of *SMN2*; **(** ***g*** **)** *NAIP* gene lacking exon13; **(** ***h*** **)** Hybrid *SMN* gene in which exon 8 of *SMN1* was converted to exon 8 of *SMN2*.

All SMA patients had a homozygous deletion of *SMN1* exon 7. We also identified a homozygous deletion of *SMN1* exon 8 in 37 of 42 patients (88.1%); the other 5 SMA patients (11.9%) had a single copy of *SMN1* exon 8. The proportions of SMA patients with various numbers of normal *SMN2* copies were 1 copy in 2 patients (4.8%), 2 copies in 14 (33.3%), 3 copies in 24 (57.1%) and 4 copies in 2 (4.8%). The proportions of SMA patients with various numbers of normal *NAIP* were 0 copies in 4 patients (9.5%), 1 copy in 26 (61.9%) and 2 copies in 12 (28.6%). In addition, 10 patients (23.8%) had an *NAIP* gene lacking exon 5 (pattern *a* in Figure 2), and 1 patient (2.4%) had an *SMN1* gene lacking exon 7 (pattern *b* in Figure 2). *SMN2* genes lacking partial sequences were also found in SMA patients. Three patients (7.1%) had an *SMN2* lacking exon 1 to exon 7 (pattern *d* in Figure 2), and two (4.8%) had an *SMN2* lacking exon 7 (pattern *c* in Figure 2). We also found hybrid *SMN* genes in SMA patients. A hybrid *SMN* gene in which exon 8 of *SMN2* was converted to exon 8 of *SMN1* (pattern *e* in Figure 2) was found in 3 patients (7.1%), and a hybrid *SMN* gene in which exon 7 of *SMN1* was converted to exon 7 of *SMN2* (pattern *f* in Figure 2) was found in 1 (2.4%). MLPA probe copy numbers of 42 SMA patients are in Table 1 and inferred copy numbers are in Table 2. Abnormal gene structures are in Figure 2.

**Table 1:** **The MLPA probe copy numbers of SMA-related genes in 42 Chinese SMA patients**

		**MLPA probe copy number**									
	**Total**	***SMN1*** **/** ***SMN2***				***SMN1***		***SMN2***		***NAIP***	
**Group**	**N = 42**	**Exon1**	**Exon4**	**Exon6**	**Exon8**	**Exon7**	**Exon8**	**Exon7**	**Exon8**	**Exon13**	**Exon5**
1	3(7.1%)	2	2	2	2	0	0	2	2	1	1
2	3(7.1%)	2	2	2	2	0	0	2	2	1	0
3	1(2.4%)	2	2	2	2	0	1	2	1	1	0
4	1(2.4%)	2	2	2	2	0	0	2	2	2	1
5	1(2.4%)	3	3	3-4	3	0	0	3	3	2	1
6	1(2.4%)	3	3	3-4	3	0	0	3	3	2	2
7	4(9.5%)	3	3	3	3-4	0	0	3	3	2	2
8	2(4.8%)	4	4	4	4	0	0	4	4	2	2
9	1(2.4%)	3	3-4	3	3	0	1	2	2	2	1
10	2(4.8%)	3	3-4	3-4	3	0	0	2	3	2	2
11	9(21.3%)	3	3-4	3	3-4	0	0	3	3	2	1
12	6(14.2%)	3	3	3	3	0	0	3	3	1	1
13	1(2.4%)	3	3	3	3	0	1	3	2	1	1
14	1(2.4%)	2	2	2	2	0	1	2	1	1	1
15	1(2.4%)	2-3	2-3	3	4	0	0	3	4	2	2
16	2(4.8%)	2-3	3	3	3-4	0	0	3	4	2	1
17	2(4.8%)	1-2	2	2	2	0	0	2	2-3	2	2
18	1(2.4%)	2-3	2-3	3	3	0	1	3	2	2	1

**Table 2:** **Copy numbers of SMA-related genes in 42 Chinese SMA patients determined by MLPA**

		**Total**	**Gene copy number**				
**Pattern**	**Group**	**N = 42**	**Normal** ***SMN1*** **gene**	**Normal** ***SMN2*** **gene**	**Normal** ***NAIP*** **gene**	**Hybrid** ***SMN*** **gene**	**Gene lacking partial sequences**
A	1	3(7.1%)	0	2	1	0	0
B	2	3(7.1%)	0	2	0	0	1^*a*^
C	3	1(2.4%)	0	1	0	1^*e*^	1^*a*^
D	4	1(2.4%)	0	2	1	0	1^*a*^
E	5	1(2.4%)	0	3	1	0	1^*a*^
F	6/7	5(11.9%)	0	3	2	0	0
G	8	2(4.8%)	0	4	2	0	0
H	9	1(2.4%)	0	2	1	0	1^*a*^ and 1^*b*^
I	10	2(4.8%)	0	2	2	0	1^*c*^
J	11	9(21.3%)	0	3	1	0	1^*a*^
K	12	6(14.2%)	0	3	1	0	0
L	13	1(2.4%)	0	2	1	1^*e*^	0
M	14	1(2.4%)	0	1	1	1^*e*^	0
N	15	1(2.4%)	0	3	2	0	1^*d*^
O	16	2(4.8%)	0	3	1	0	1^*a*^ and 1^*d*^
P	17	2(4.8%)	0	2	2	0	0
Q	18	1(2.4%)	0	2	1	1^*f*^	1^*a*^

^*a*^:*NAIP* gene lacking exon 5, ^*b*^:*SMN1* gene lacking exon7, ^*c*^:*SMN2* gene lacking exon7, ^*d*^:*SMN2* gene lacking exon 1 to exon 7, ^*e*^:Hybrid *SMN* gene in which exon 8 of *SMN2* gene was converted to exon 8 of *SMN1*, ^*f*^:Hybrid *SMN* gene in which exon 7 of *SMN1* gene was converted to exon 7 of *SMN2*.

### Gene copy number and structure analysis of unrelated healthy individuals

Using MLPA assays, 212 healthy Chinese participants were analyzed. We found that 117 (55.2%) had normal gene copy numbers for *SMN1*/*SMN2/NAIP* (2/2/2) and 54 (25.5%) had different gene copies numbers for *SMN1*/*SMN2 NAIP* (2/1/2). The proportions of the 212 individuals with different copy numbers for the normal *SMN1* gene were 1 copy in 4 individuals (1.9%), 2 copies in 203 (95.7%) and 3 copies in 5 (2.4%). The proportions of the 212 with different gene copy numbers for normal *SMN2* were 0 copies in 10 (4.7%), 1 copy in 70 (33.0%), 2 copies in 130 (61.4%) and 3 copies in 2 (0.9%). The proportions of the 212 with different gene copies for normal *NAIP* were 1 copy in 15 (7.1%) and 2 copies in 197 (92.9%). Genes lacking partial sequences were also found in healthy participants. Five (2.4%) had an *NAIP* gene lacking exon 5 (pattern *a* in Figure 2) and 27 (12.7%) had an *NAIP* gene lacking exon 13 (pattern *g* in Figure 2). One person appeared to have an *SMN1* gene lacking exon 7 (pattern *b* in Figure 2) and an *SMN2* gene lacking exon 7 (pattern *c* in Figure 2). Three types of hybrid *SMN* genes were found in 6 of the healthy participants. Four (1.9%) had a hybrid *SMN* gene in which exon 8 of *SMN2* was converted to exon 8 of *SMN1* (pattern *e* in Figure 2). Two (0.9%) had a hybrid *SMN* gene in which exon 8 of *SMN1* was converted to exon 8 of *SMN2* (pattern *h* in Figure 2). MLPA results for 212 healthy individuals are in Table 3 and inferred gene copy numbers are in Table 4. Abnormal gene structures are in Figure 2.

**Table 3:** **The MLPA probe copy numbers of SMA-related genes in 212 Chinese healthy individuals**

		**MLPA probe copy number**									
	**Total**	***SMN1*** **/** ***SMN2***				***SMN1***		***SMN2***		***NAIP***	
**Group**	**N = 212**	**Exon1**	**Exon4**	**Exon6**	**Exon8**	**Exon7**	**Exon8**	**Exon7**	**Exon8**	**Exon13**	**Exon5**
1	117(55.2%)	4-5	4-5	4	4-5	2	2	2	2	2	2
2	1(0.5%)	4	4	4	4	2	3	2	1	2	2
3	1(0.5%)	4	4-5	4	4	2	1	2	3	2	2
4	54(25.4%)	3-4	3-4	3	3-4	2	2	1	1	2	2
5	1(0.5%)	3	3	3	3	2	3	1-2	0	2	2
6	7(3.3%)	3-4	3	3-4	3-4	2	2	1	1	2	3
7	1(0.5%)	3-4	3-4	3-4	3	2	2	1	1	2	4
8	4(1.9%)	2	2	2	2	2	2	0	0	1	2
9	5(2.3%)	2	2-3	2-3	2-3	2	2	0	0	1	3
10	1(0.5%)	3	3	3-4	3-4	2	2	1	1	1	1
11	5(2.3%)	4	4-5	4	4	2	2	2	2	2	3
12	1(0.5%)	4	4-5	4	4-5	2	1	2	3	2	3
13	2(0.9%)	4	4-5	4	4-5	2	3	2	1	2	3
14	2(0.9%)	4	4	4	4	3	3	1	1	2	2
15	3(1.4%)	4	4-5	4	4-5	2	2	2	2	2	1
16	2(0.9%)	4-5	5	5	4-5	2	2	3	3	2	2
17	1(0.5%)	4	4-5	4	5	1	2	1	2	2	2
18	1(0.5%)	4-5	5	5	4-5	3	3	2	2	2	3
19	1(0.5%)	4-5	5	4-5	5	3	3	2	2	2	1
20	1(0.5%)	3	3-4	3-4	3-4	1	1	2	2	2	1
21	1(0.5%)	4	4	4	4	3	3	1	1	2	3

**Table 4:** **Copy numbers of SMA-related genes in 212 Chinese healthy individuals determined by MLPA**

		**Total**	**Gene copy number**				
**Pattern**	**Group**	**N = 212**	**Normal** ***SMN1*** **gene**	**Normal** ***SMN2*** **gene**	**Normal** ***NAIP*** **gene**	**Hybrid** ***SMN*** **gene**	**Gene lacking partial sequences**
A	1	117(55.2%)	2	2	2	0	0
B	2	1(0.5%)	2	1	2	1 ^*e*^	0
C	3	1(0.5%)	1	2	2	1 ^*h*^	0
D	4	54(25.5%)	2	1	2	0	0
E	5	1(0.5%)	2	0	2	1 ^*e*^	0
F	6/7	8(3.8%)	2	1	2	0	1 ^*g*^
G	8/9	9(4.2%)	2	0	1	0	1 ^*g*^
H	10	1(0.5%)	2	1	1	0	0
I	11	5(2.3%)	2	2	2	0	1 ^*g*^
J	12	1(0.5%)	1	2	2	1 ^*h*^	1 ^*g*^
K	13	2(0.9%)	2	1	2	1 ^*e*^	1 ^*g*^
L	14	2(0.9%)	3	1	2	0	0
M	15	3(1.4%)	2	2	1	0	1 ^*a*^
N	16	2(0.9%)	2	3	2	0	0
O	17	1(0.5%)	1	1	2	0	1 ^*b*^ and 1 ^*c*^
P	18	1(0.5%)	3	2	2	0	1 ^*g*^
Q	19	1(0.5%)	3	2	1	0	1 ^*a*^
R	20	1(0.5%)	1	2	1	0	1 ^*a*^
S	21	1(0.5%)	3	1	2	0	1 ^*g*^

^*a*^:*NAIP* gene lacking exon 5, ^*b*^:*SMN1* gene lacking exon 7, ^*c*^:*SMN2* gene lacking exon 7, ^*e*^:Hybrid *SMN* gene in which exon 8 of *SMN2* was converted to exon 8 of *SMN1*, ^*g*^:*NAIP* gene lacking exon13, ^*h*^:Hybrid *SMN* gene in which exon 8 of *SMN1* was converted to exon 8 of *SMN2*.

### Differences in distribution of participants by ***SMN2*** and ***NAIP*** gene copy numbers

We examined the distribution of different copy numbers for the normal *SMN2* and *NAIP* genes in SMA patients and normal individuals. Distributions by copy number for normal *SMN2* were significantly different in SMA patients compared to participants without SMA (*P* < 0.001) (Table 5). Distributions by copy number for normal *NAIP* were significantly different for SMA patients compared to healthy participants (*P* < 0.001) (Table 6).

**Table 5:** **Comparison of the distribution of** ***SMN2*** **copy number in SMA patients and healthy individuals**

**Number of normal** ***SMN2***	**SMA patients (N = 42)**	**Healthy individuals (N = 212)**	***P***
0	0 (0%)	10 (4.7%)	
1	2 (4.8%)	70 (33.1%)	
2	14 (33.3%)	130 (61.3%)	<0.001
3	24 (57.1%)	2 (0.9%)	
4	2 (4.8%)	0 (0%)

**Table 6:** **Comparison of the distribution of** ***NAIP*** **copy number in SMA patients and healthy individuals**

**Number of normal** ***NAIP***	**SMA patients (N = 42)**	**Healthy individuals (N = 212)**	***P***
0	4 (9.5%)	0 (0%)	
1	26 (61.9%)	15 (7.1%)	<0.001
2	12 (28.6%)	197 (92.9%)	

## Discussion

In this study, the copy numbers for *SMN1*, *SMN2* and *NAIP* were determined in Chinese SMA patients and healthy controls using MLPA assays and plausible gene structures were inferred. We found that 37 (88.1%) people with SMA had deletions in both exon 7 and exon 8 of *SMN1* and the other 5 SMA patients (11.9%) had a single copy of *SMN1* exon 8. These results were similar to several other studies in different populations [14,15]. Chen et al. reported that a distribution for *SMN2* copy numbers in 94 Chinese SMA patients of 5 copies in 1 patient (1.1%), 4 copies in 24 (25.5%), 3 copies in 47 (50%) and 2 copies in 22 (23.4%). No patients with 0 or 1 copy of *SMN2* were found in their population [16]. In addition, Qu et al. reported that a distribution for *SMN2* copy numbers in 232 Chinese SMA patients of 4 *SMN2* copies in 13 patients (5.6%), 3 copies in 153(65.9%), 2 copies in 66(28.5%) and no patients having only 0 or 1 copy of *SMN2* [8]. In our results, the proportion of SMA patients with a single normal *SMN2* copy was 4.8% and no SMA patients completely lacked copies of *SMN2*. Although two SMA patients had a single normal copy of *SMN2*, they also had a hybrid *SMN* gene. These findings indicated that the proportion of SMA patients with 0 or 1 copy of *SMN2* was low in Chinese SMA patients. Similar to the previous report, 2 patients (4.8%) had 4 *SMN2* copies, and no patients had 5 or 6 *SMN2* copies. The results revealed that the proportion of SMA patients with more than 4 *SMN2* copies was low in Chinese population. Most of Chinese SMA patients had 2 or 3 *SMN2* copies. The distribution of normal copy numbers of *NAIP* in 42 SMA patients were: homozygous deletion (9.5%), 1 copy (61.9%), and 2 copies (28.6%). This result is similar to data reported by another two studies of Chinese SMA patients [8,17]. Using conversions between *SMN1* and *SMN2* in exons 7 and 8, six possible hybrid *SMN* genes were determined [10]. The hybrid *SMN* genes of patterns *e* and *f* have been reported in Chinese SMA patients [10,16]. In addition, four types of genes lacking partial sequences were found in SMA patients in this study. Ten SMA patients had an *NAIP* gene lacking exon 5, and this type (pattern *a* in Figure 2) of deletion was also found in several studies [7,18,19]. Three patients had an *SMN2* lacking exon 1 to exon 7 (pattern *d* in Figure 2), and two had an *SMN2* lacking exon 7 (pattern *c* in Figure 2). We also inferred that one patient had an *SMN1* lacking exon 7 (pattern *b* in Figure 2). For all we know, this study is the first report of three types of *SMN* (pattern *b*, *c*, *d*) lacking partial sequence.

The SMA-related genes of 212 Chinese healthy individuals were analyzed using MLPA assays. Zhu et al. reported that 1% of healthy Chinese individuals have four *SMN1* copies [2]. However, no individuals with 4 copies of *SMN1* were found in our study. In addition, we identified 4 participants with a single normal *SMN1* copy, for a carrier frequency of 1.9% in our study. The reported frequency of SMA carriers is 2.4% in the general Chinese population [2,16], therefore, our carrier frequency was lower. In contrast to the SMA patients, no homozygous deletions of *NAIP* were found in healthy Chinese participants. In addition to the hybrid *SMN* gene *e* in SMA patients, the hybrid *SMN* gene in which exon 8 of *SMN1* was converted to exon 8 of *SMN2* (pattern *h* in Figure 2) was found in healthy participants; this type of the hybrid *SMN* gene has been reported previously [10,20]. Four types of genes lacking partial sequences (pattern *a*, *b*, *c*, *g* in Figure 2) were found in healthy individuals of our study. The proportion of healthy individuals (2.4%) with an *NAIP* gene lacking exon 5 was lower than for SMA patients (23.8%). This result might indicate that deletion of exon 5 in *NAIP* is involved in the molecular basis of SMA. Of the healthy participants, 27 (12.7%) had an *NAIP* gene with an exon 13 deletion, and this type of *NAIP* gene was not found in SMA patients. One SMA carrier had a ratio of 1:2 for exon 7 and 8 of *SMN1* and a ratio of 1:2 for exon 7 and 8 of *SMN2*. We hypothesized that she had an *SMN1* lacking exon 7 (pattern *b* in Figure 2) and an *SMN2* lacking exon 7 (pattern *c* in Figure 2).

The distributions of different copy numbers for normal *SMN2* were compared between Chinese SMA patients and healthy individuals and distributions were significantly different (*P* < 0.001) between SMA patients and people without SMA (Table 5). Similarly, in a study of 108 SMA patients and 22 healthy controls, Crawford et al. found that the *SMN2* copy number was significantly lower in control subjects [21]. Thus, SMA patients appeared to be prone to having more *SMN2* copies than the controls. Because of the lack of SMN protein, fewer copies of *SMN2* decrease the likelihood of survival to birth. This might explain the difference in the distribution of people with different *SMN2* copy numbers. We also analyzed the distribution of copy numbers of normal *NAIP* in SMA patients and people without SMA and found significant differences (*P* < 0.001) (Table 6). Compared with healthy people, SMA patients had fewer *NAIP* copies. *NAIP* modifies the clinical phenotypes of SMA patients. Patients with fewer copies of *NAIP* might have more severe clinical symptoms of SMA [6,7]. This might be why that distribution of *NAIP* copies was significantly different between people with and without SMA. The *NAIP* gene locus is near the *SMN1* gene locus, and both are located at 5q13.2. We determined that some SMA patients had a large deletion in the *SMN1* and *NAIP* loci. This could partially explain why SMA patients had fewer *NAIP* copies than people without SMA.

Currently, *SMN1* is recognized as an SMA causing gene. In contrast, the *SMN2* and *NAIP* have been characterized as a modifying factor of the clinical severity of SMA. So, the clinical diagnosis and prenatal diagnosis of SMA are mainly based on the identification of *SMN1* copy numbers and the situation of the *SMN1* gene exon7 [22-24]. The copy numbers of *SMN2* and *NAIP* were used to evaluate the clinical phenotype of SMA patients [25,26]. According to our results, the differences between the SMA patients and healthy controls were not only the distribution of SMA-related genes, the gene structures were also different. Moreover, the proportions of abnormal gene structures were high in both SMA patients (36%) and healthy controls (17%). Our data revealed that only confirming the gene copy numbers may not be sufficient to the clinical diagnosis, prenatal diagnosis, phenotype evaluation and carrier screening of SMA. The abnormal gene structures should be taken into account in the clinical diagnosis of SMA. So the analyses of SMA-related gene structures were also important to the molecular diagnostics of SMA patients and carriers.

At present, most of previous studies about Chinese SMA disease only focused on SMA patients or healthy population. There were few articles about comparison and analysis of SMA-related genes and gene structures in the Chinese people with and without SMA. In this study, we compared copy numbers of *SMN1*, *SMN2* and *NAIP* between Chinese SMA patients and healthy controls and inferred gene structures. We summarized the subdivision of SMA-related gene patterns (Tables 2 and 4) based on the MLPA technique. The genetic characteristics would be helpful for the further identification of the clinical subdivision of SMA patients. There are limitations of our study. All gene copy numbers and gene structures were inferred from MLPA results, so we didn’t know the detailed haplotype of each participant. Therefore, further investigations are needed.

## Conclusions

In conclusion, we analyzed and compared gene copy numbers and gene structures in Chinese SMA patients and healthy individuals. For the first time three types of *SMN* lacking partial sequence were found. The distributions of different copy numbers for normal *SMN2* and *NAIP* were significantly different between SMA patients and healthy controls. The gene structures of *SMN* and *NAIP* were also different between the SMA patients and healthy controls. Further studies are required to address the molecular mechanisms and clinical diagnoses of SMA.

## Abbreviations

MLPA: Multiple ligation-dependent probe amplification
*NAIP*: Neuronal apoptosis inhibitory protein
SMA: Spinal muscular atrophy
*SMN*: Survival motor neuron
